# Metabolic response of porcine colon explants to in vitro infection by *Brachyspira hyodysenteriae*: a leap into disease pathophysiology

**DOI:** 10.1007/s11306-017-1219-6

**Published:** 2017-05-30

**Authors:** Thijs Welle, Anna T. Hoekstra, Ineke A. J. J. M. Daemen, Celia R. Berkers, Matheus O. Costa

**Affiliations:** 1 Department of Farm Animal Health, Faculty of Veterinary Medicine, Utrecht University, Yalelaan 7, 3584 CL Utrecht, The Netherlands; 2 Biomolecular Mass Spectrometry and Proteomics, Bijvoet Center for Biomolecular Research, Utrecht University, Utrecht, The Netherlands

**Keywords:** *Brachyspira*, Swine dysentery, In vitro organ culture, Host-pathogen interactions, Pathophysiology, Nitric oxide

## Abstract

**Introduction:**

Swine dysentery caused by *Brachyspira hyodysenteriae* is a production limiting disease in pig farming. Currently antimicrobial therapy is the only treatment and control method available.

**Objective:**

The aim of this study was to characterize the metabolic response of porcine colon explants to infection by *B. hyodysenteriae*.

**Methods:**

Porcine colon explants exposed to *B. hyodysenteriae* were analyzed for histopathological, metabolic and pro-inflammatory gene expression changes.

**Results:**

Significant epithelial necrosis, increased levels of l-citrulline and IL-1α were observed on explants infected with *B. hyodysenteriae*.

**Conclusions:**

The spirochete induces necrosis in vitro likely through an inflammatory process mediated by IL-1α and NO.

**Electronic supplementary material:**

The online version of this article (doi:10.1007/s11306-017-1219-6) contains supplementary material, which is available to authorized users.

## Introduction

Swine dysentery is an infectious disease that affects most pork-producing countries in the world. The etiological agent associated with it is the anaerobic, gram-negative spirochete *Brachyspira hyodysenteriae*. Swine dysentery typically manifests as severe colitis, concomitant with mucoid and haemorrhagic diarrhea in grower and finisher pigs (Alvarez-Ordonez et al. 2013). It is a production-limiting disease affecting animal performance, increasing costs with antimicrobial therapy and carcass variability at slaughter. Currently, control of swine dysentery relies solely on biosecurity and antibiotic therapy, usually in the form of continual in-feed medication, as no commercial vaccine is available for swine dysentery. The mechanisms by which *B. hyodysenteriae* induces tissue damage leading to epithelial necrosis, colitis and luminal bleeding are not clear. This knowledge gap likely contributes to the lack of alternatives to antibiotic therapy for prevention of clinical disease. Furthering our understanding of disease pathophysiology will help improve our ability to treat and control the disease, and may contribute to the development of preventive tools in the future.

The objective of this study was to profile the metabolites in swine colon explants after in vitro exposure to *B. hyodysenteriae* for 8 h.

## Materials and methods

### Pathogen isolation and characterization

*Brachyspira hyodysenteriae* isolation from fecal samples, propagation and characterization was performed as previously described for other *Brachyspira* spp. (Rubin et al. 2013). Briefly, fresh feces were collected from a naturally infected pig with mucohaemorrhagic diarrhea and transported on ice for isolation. A sterile loop was used to inoculate BJ agar and, after 72 h of anaerobic incubation at 37 °C, patches of strong β-haemolysis were observed from which motile spirochetes could be seen on phase microscopy. This procedure was repeated two times to obtain an isolated spirochete culture. Areas of clear, strong β-haemolysis were sampled and DNA was extracted using a commercial kit (DNeasy Blood and Tissue Kit; QIAGEN, Hilden, Germany). To speciate the isolate, a genus specific PCR targeting the NADH-oxidase (*nox*) gene (Rohde et al. 2002) was performed and amplicons were submitted for sequencing. Amplicon (939 bp) BLAST analysis revealed 99% similarity to other *B. hyodysenteriae* isolates (accession number).

### Colon segment collection and explant preparation

This article does not contain any studies with human participants or animals performed by any of the authors. Three piglets between 5 and 7 weeks of age, fed commercial diet and free of gastrointestinal clinical signs were euthanized for reasons unrelated to this study. Pigs were sedated with 8 mg/kg IM of azaperone (Stresnil, Lifarma BV, Baexem, The Netherlands) and euthanized by pentobarbital overdose (intracardiac, Euthasol 20%, AST farma BV, Oudewater, The Netherlands), according to institutional guidelines for the care and use of animals. Tissue collection and preparation for culture was performed as previously described (Costa et al. 2016). Immediately after euthanasia an 8 cm segment of spiral colon (apex) was surgically excised through a 10 cm incision along the *linea alba*. The colon segment was placed in 30 ml of refrigerated Dulbecco phosphate buffered saline (DPBS without Ca^2+^ and Mg^2+^, 0.1 M, pH 7.0) supplemented with an antibiotic mix selective for *Brachyspira* spp. (200 µg/ml spectinomycin, 6.25 µg/ml vancomycin, 6.25 µg/ml colistin, 25 µg/ml spiramycin, 12.5 µg/ml rifampicin). Within 1 h of euthanasia, colonic serosa was surgically separated from the mucosa on a refrigerated surface embedded in the same solution used for transport. The mucosa from each colon segment was then divided into 28 explants of 1.5 cm x 1.5 cm, which were placed on individual 3 cm x 3 cm x 0.75 cm agar blocks (1% w/v in distilled water) kept in dishes (100 mm x 15 mm) containing 8 ml of culture media. Next, dishes were placed in a modular incubator chamber and it was gassed for 2 min at 10 l/min with a 99% O_2 −_ 1% CO_2_ gas mix (Medical Carbogeen, Linde Healthcare Benelux, Eindhoven, The Netherlands). This setup was incubated at 37 °C. Culture media consisted of keratinocyte basal media (KBM-gold Keratinocyte, Lonza Benelux BV, Breda, The Netherlands) supplemented with 1.5 mM Ca^2+^ and the above mentioned *Brachyspira* spp. selective antibiotic mix.

### Inoculum preparation

Agar pieces from cultures of the isolate described above (1 cm x 1 cm) displaying strong β-haemolysis were used to inoculate glass vials containing 25 ml of JBS broth. Vials were placed on a shaker (160 rpm) and incubated anaerobically at 37 °C for 48 h. Immediately before infecting the explants, culture vials were retrieved from the incubator and an aliquot was collected for verification of bacterial activity and quantification by quantitative PCR (kept frozen at −80 °C until processing). Phase contrast microscopy was used to visually assess spirochete motility as an indicator of viability. 1 ml of culture broth containing active, motile spirochetes was centrifuged for 10 min at 2500×*g*. The supernatant was discarded and the pellet was resuspended in 1 ml of sterile PBS with above mentioned antibiotic solution. For preparation of control inoculum, 25 ml of sterile JBS broth was incubated, centrifuged and resuspended as the infected inoculum. Before inoculation, lipopolysaccharide (LPS, *E. coli* O:127 B:8, Sigma, Zwijndrecht, The Netherlands) was diluted in sterile PBS (10 µg/ml). All inocula were supplemented with the same antibiotic mix selective for *Brachyspira* spp.

### Infection trial

In this study, three groups were investigated: PBS group (negative control), where explants were inoculated with the control inoculum; LPS (positive control), where explants received the previously mentioned LPS preparation; and* B. hyo*, where explants received active, pure cultures of *B. hyodysenteriae*. Before inoculation, explants were randomly assigned to one of the three groups (n = 9/group/pig). Each explant received a polypropylene inoculation ring (0.5 cm diameter x 1 cm height) attached to its mucosal (luminal) aspect by an innocuous, medical-grade silicone adhesive (Kwik-Cast, World Precision Instruments, Sarasota, Fla., USA). The ring was used to prevent inoculum spillage beyond the mucosal side. Each explant receiving a total of 100 µl of inoculum, independent of group. After inoculation, explants were co-incubated with inocula for 8 h in the conditions described above for explant culture. At this time, 2 explants/group/pig were snap frozen in liquid nitrogen, and stored at −80 °C until qPCR analysis. Other 1 explant/group/pig was fixed in Carnoy’s solution (60% absolute ethanol, 30% chloroform and 10% glacial acetic acid v/%.) for 2 h, and then moved to a 70% ethanol, 30% distilled water solution until processing for histopathology. The remaining 6 explants/group/pig were also snap frozen in liquid nitrogen and stored in −80 °C until further processing for metabolite extraction and analysis. One extra colon section per pig was processed up to inoculation, and then immediately fixed in Carnoy’s solution to be used as a technique control (0 h samples).

### H&E stained sections

Carnoy’s fixed tissue was routinely processed in paraffin, sectioned and stained using hematoxylin and eosin (H&E). From each explant (n = 9), four 4 µm sections were prepared in order to have representative samples of the entire explant length. An examiner blinded to the identity of samples carried out the analysis under optical microscopy at 20× magnification. A semi-quantitative scoring system was applied, which evaluated epithelial cells in regards to nuclei karyopyknosis or karyorrhexis, loss of cell membrane delimitation and increased cytoplasmic vacuolization. Score 0: 0–20% of crypt cells were necrotic; score 1: 21–30% of crypt cells were necrotic; score 2: 31–70% of crypt cells were necrotic; score 3: 71–100% of crypt cells were necrotic. The entire section length was evaluated.

### Quantitative PCR assays

Analysis of mRNA levels from explants targeted the GAPDH, interleukin-1α (IL-1α), interleukin-8 (IL-8), interferon gamma (IFN-γ), tumor necrosis factor alpha (TNF-α) genes. Assays were conducted as previously described (Costa et al. 2016). Briefly, total RNA was extracted from RNAlater fixed samples and cDNA was assembled using commercially available kits (RNeasy minikit, Qiagen NV, Venlo, The Netherlands, iScript cDNA synthesis kit, Bio-Rad, Veenendaal, The Netherlands). All quantitative PCR reactions were conducted on a Bio-Rad MyiQ single color Real Time Detection System (Bio-Rad, Veenendaal, The Netherlands). Each 25 µl reaction contained 1 × IQ SYBR Green Supermix (Bio-Rad, Veenendaal, The Netherlands), forward and reverse primers (0.8 µM each), and 1 µl of cDNA template. Reactions were incubated at 95 °C for 3 min, followed by 40 cycles of (30 s at 95 °C, 30 s at 64 °C and 30 s at 72 °C) and a final extension step of 5 min at 72 °C. cDNA template used for all reactions was normalized to the lowest amount detected across all samples. All reactions were run in duplicate, and both extraction negatives and no-template controls were included for each assay. Reaction duplicates that differed by more than 1 Cq value were repeated. Amplification efficiencies for all PCR assays were between 95 and 103%.

Quantification of *B. hyodysenteriae* from pure cultures used a genus-specific quantitative PCR protocol adapted from a previously described technique (Rubin et al. 2013).

### Metabolite extraction and quantification

Prior to liquid chromatography-mass spectrometry (LC-MS) analysis, explants were homogenized using a liquid nitrogen cooled mini-mortar (Bel-Art Products). When the tissue was reduced to fine powder, metabolites were extracted by adding 300 μl of methanol/acetonitrile/ULC/MS grade water (Biosolve BV, Valkenswaard, The Netherlands) (2:2:1) lysis buffer and sonicating in a Bioruptor Plus (Diagenode, Liège, Belgium) at high power with eight sonication cycles (15 s. ON, 60 s. OFF). After sonication, samples were shaken for 10 min at 4 °C and centrifuged for 15 min at 14,000*g* and 4 °C. Supernatants were collected and transferred into a separated vial and stored at −80 °C awaiting subsequent analysis. Liquid chromatography-mass spectrometry analysis was performed using an Exactive mass spectrometer (Thermo Scientific), coupled to a Dionex Ultimate 3000 auto sampler and pump (Thermo Scientific). The mass spectrometry operated in polarity-switching mode with spray voltages of 4.5 and −3.5 kV. Metabolites were separated using a Sequant ZIC-pHILIC column (2.1 × 150 mm, 5 mm, guard column 2.1 × 20 mm, 5 mm; Merck) using a linear gradient of acetonitrile and eluent A 20 mM (NH_4_)_2_CO_3_, 0.1% NH_4_OH in ULC/MS grade water (Biosolve BV, Valkenswaard, The Netherlands) and a flow rate of 150 µl/min. Peak intensities were normalized on median ion count using IDEOM software (Creek et al. 2012). Metabolites were identified and quantified using LCquan software (Thermo Scientific) on the basis of exact mass within 5 ppm and further validated by concordance with retention times of commercially available standards.

### Statistical analysis

Histopathology and gene expression data analyses were carried out using SPSS version 24 (SPSS Inc., Chicago, USA) and R Studio version 0.99.893 (RStudio Team 2015), respectively. Necrosis scores were compared using Kruskall–Wallis test adjusted by the Bonferroni correction for multiple tests followed by Dunn’s *post-hoc* test. This data is presented as median ± interquartile range. Explant mRNA levels were compared using the MCMC.qpcr (version 1.2.2) R package (Matz et al. 2013). This method applies generalized linear mixed model with Poisson-lognormal errors and a Bayesian Marco Chain Monte Carlo sampling scheme to infer fold changes in mRNA levels in response to fixed factors and random differences attributable to technical replicates. Gene expression data were graphed as mean fold-change (±standard error of the mean) using PBS samples as a reference group.

Metabolomics data were log-transformed, scaled by each variable standard deviation, and analyzed by multivariate PCA, orthogonal PLS-DA, and univariate ANOVA with pairwise comparisons and *post-hoc* correction for multiple hypothesis testing using Fisher’s least significant difference method embedded in the MetaboAnalyst 3.0 suite (Xia et al. 2015). The latter was also used for integrated enrichment and pathway topography analysis, which employed the *Homo sapiens* library as a reference since a *Sus scrofa* library is not available.

## Results and discussion

### Increased epithelial necrosis and mucosal erosion

**Fig. 1:**
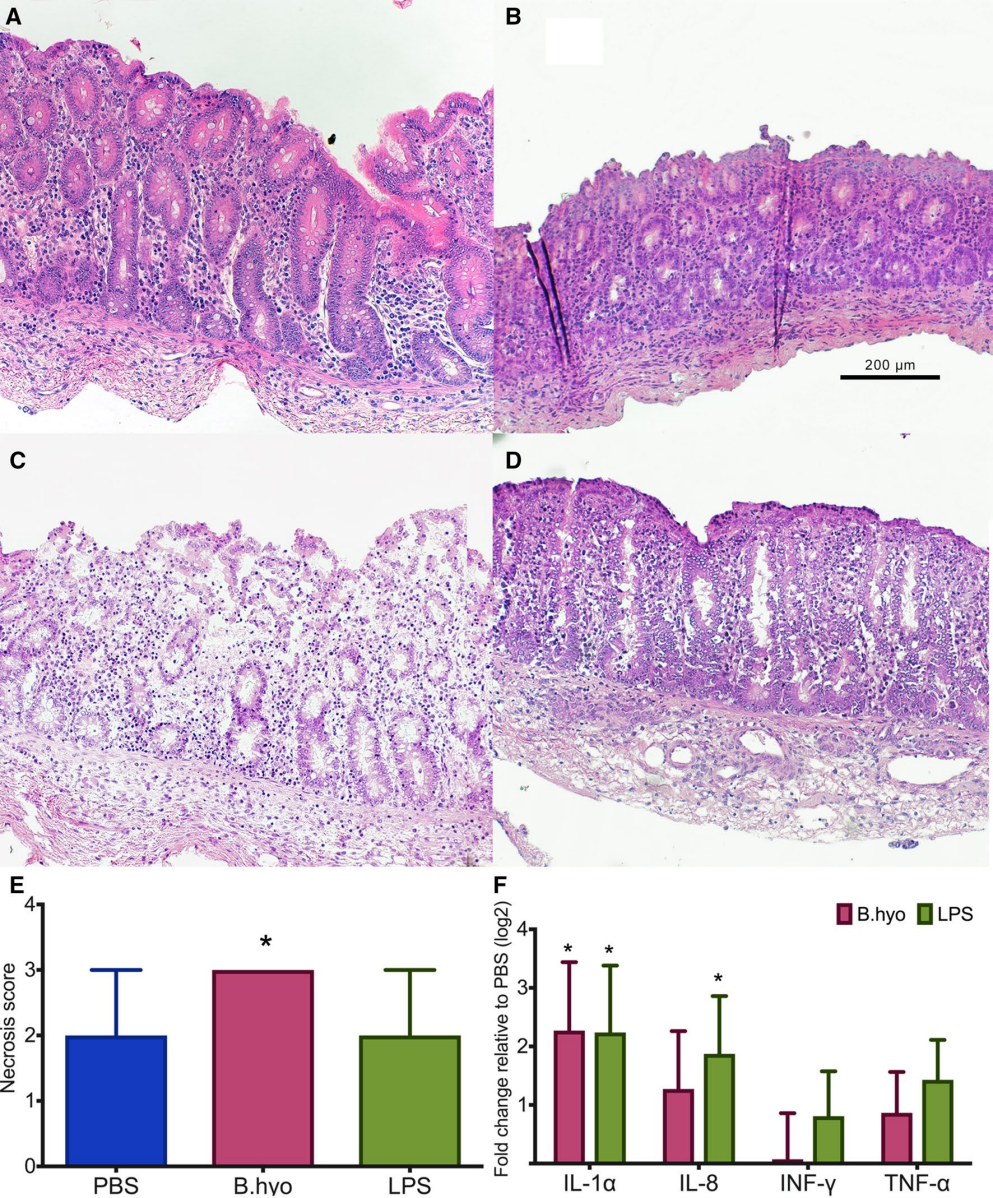
Histopathology and gene expression data summaries. Explants were fixed in Carnoy’s solution. **a** Typical section of control explant sampled at 0 h. **b** PBS-inoculated explant (score 1). **c** Representative* B. hyo* explant section (score 3), displaying significant epithelial necrosis and sloughing off epithelial cells. **d** LPS group explant section (score 2). **e** *Bars* represent median (±interquartile range) epithelial necrosis scores for each inoculation group (n = 12 sections/group). *Star* denotes statistical difference between* B. hyo* and both other groups (*P* < 0.05). **f** Each *bar* shows mean fold-change (±standard error of the mean) in mRNA levels from a given group relative to the PBS group (n = 6/group). *Star* denotes significant mRNA levels difference between a given group and PBS (*P* ≤ 0.05)

Explants from* B. hyo* group received an average of 4.50 × 10^6^ genome equivalents of *B. hyodysenteriae* upon inoculation. A total of 36 H&E stained explant sections were evaluated and a score difference between groups was observed (*P* = 0.015). *Post-hoc* analysis revealed that *B. hyodysenteriae* infected group had greater scores than both PBS and LPS groups (*P* = 0.037 and 0.04, respectively, Fig. 1a). All 0-hour samples received a score of 0.

In this study, exposure of explants to live *B. hyodysenteriae* cells resulted in a greater number of necrotic cells than LPS or PBS. In vivo, the earliest lesion detectable in clinical swine dysentery cases is mucosal intercellular edema, described before the onset of diarrhea but days after pigs were inoculated with the spirochete (Albassam et al. 1985). Disease progression leads to epithelial necrosis and erosion of the mucosa, exudation of fluid and necrotic cells from the exposed lamina propria and engorged superficial vessels (Hughes et al. 1977). More recently, inoculation of different *Brachyspira* strains capable of inducing mucohaemorrhagic diarrhea in a pig model revealed neutrophilic inflammation, crypt elongation and mucosal ulceration (Wilberts et al. 2014). We observed lesions that shared similar traits to what was reported in vivo, characterized by crypt cell necrosis and mucosal ulceration. In explants, the lack of physiological features such as peristalsis, for clearance of luminal mucus, and lymph and blood circulation, for metabolite removal and immune cell migration, and limited period of study may have prevented the development other of typical microscopical findings of swine dysentery.

### Pro-inflammatory cytokine profile after spirochete inoculation

Analysis of mRNA fold changes between groups revealed that both LPS and *B. hyodysenteriae* inoculated groups had increased expression of IL-1α mRNA (*P* = 0.05) when compared to the PBS group. Concomitantly, LPS exposure led to increased levels of IL-8 and TNF-α mRNA (*P* = 0.05 and *P* = 0.03, respectively). No significant difference between groups was observed for INF, even though LPS and *B. hyodysenteriae* groups had increased mRNA levels when compared to the PBS groups (Fig. 1b). The GAPDH gene, which was used as a prior in the gene expression statistical model, had an average Cq difference between PBS and both other groups of equal or less than 1 Cq across all samples.

These results show a pro-inflammatory cytokine profile after explant exposure to *B. hyodysenteriae* that resembles the description from clinical cases of swine dysentery: increased IL-1β and TNF-α during peak clinical signs (Kruse et al. 2008). The inflammatory response characterized in this study is a combination of the response built by leukocytes present in the mucosa at the time of euthanasia, the epithelium and stroma cells, and it lacks the enhancement from blood stream migrating immune cells and other immune modulators.

### Increased citrulline concentration in *B. hyodysenteriae* explants

Overall, 110 metabolites were identified from the 54 explants analyzed by LC-MS. PCA analysis revealed greater between-subject variation than between-group variation, leading to no clear group structure when plotting components (Supplementary Fig. 1a). A supervised discriminant approach revealed three different clusters, one for each inoculum group (Supplementary Fig. 1b). This group difference was further validated by a permutation test (Q^2^ = 0.82, *P* ^2^ = 0.92, 1000 permutations, *P* < 0.0001). Integrated enrichment and pathway topography analysis disclosed a set of metabolic pathways affected by explant exposure to *B. hyodysenteriae*, when compared to PBS inoculated explants (Supplementary Table 1). Among the most deeply impacted pathways are the metabolism of alanine, aspartate and glutamate, synthesis and degradation of ketone bodies, and pyruvate metabolism. Taken individually, the concentration of 21 metabolites was significantly different across groups (Supplementary Table 2). Out of these, only three metabolites were found in greater amounts in the *Brachyspira* group than the others: l-citrulline, cytosine and d-glucose 6-phosphate. This remarkable tissular accumulation of citrulline was unexpected.

**Fig. 2:**
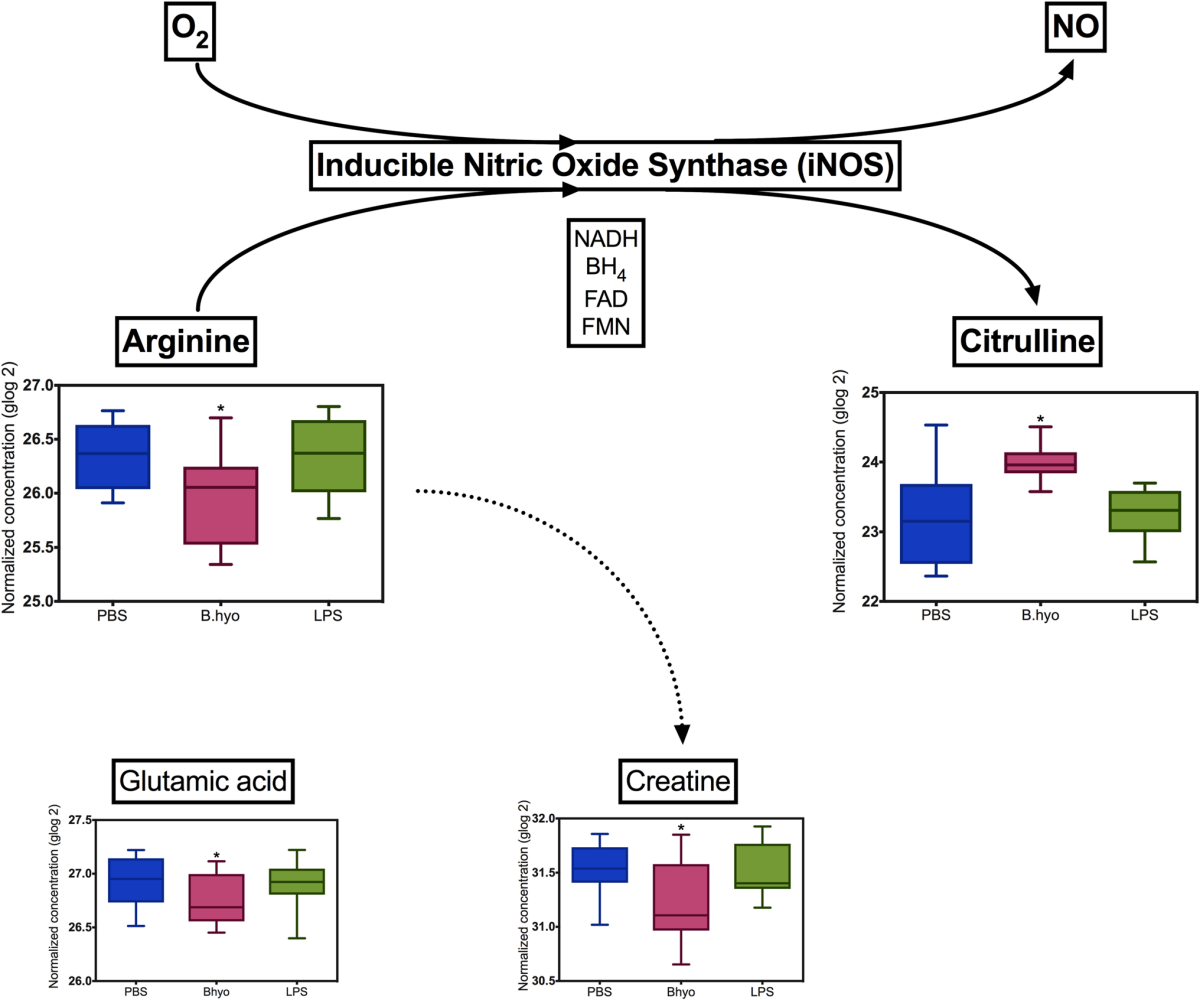
Inducible nitric oxide synthase (iNOS) catalyzed reaction. Arginine and molecular oxygen are used as substrates by iNOS in a reaction that produces NO, leaving citrulline as a by-product. NADH, BH_4_ (sapropterin), FAD and FMN (flavin mononucleotide) are co-factors. Glutamic acid is a precursor of arginine, whereas creatine is synthesized from arginine. *Dotted lines* are pathways not directly related to the synthesis of NO. Charts display transformed concentrations of metabolites obtained from explants exposed to a given inoculum for 8 h. *Bars* represent mean concentrations (5–95 percentile range) for each group (n = 18). *Star* denotes significant differences in the levels of metabolite detected between groups

Citrulline is a non-essential, non-protein forming amino acid that in higher mammals mainly participates in three metabolic pathways: arginine bio-synthesis, characterized by citrulline exchanges at whole body level; nitric oxide (NO) cycle, involved in local recycling of citrulline; and the complete urea cycle, which occurs in the liver. Under physiological conditions, most of the citrulline is found in the liver and the intestines (Curis et al. 2005). Enzymatic production of NO is catalyzed by nitric oxide synthase (NOS). Out of the three NOS isoforms described, only one is Ca^2+^-independent and inducible by bacterial toxins and cytokines (iNOS), leading to endothelial hyperpermeability and inflammation (Witthoft et al. 1998; Chavez et al. 1999). Citrulline is the main by-product of enzymatic NO synthesis, which uses d or l-arginine as substrate (Nagase et al. 1997; Singer et al. 1996). Interestingly, we found significantly lower concentrations of three amino acids related to the NO cycle in *Brachyspira*-exposed explants, when compared to the other two groups. The amino acids were l-arginine (NO substrate), creatine (alternative product of arginine metabolism) and glutamic acid (which can fuel the urea cycle through the synthesis of aspartic acid, Fig. 2). NO exerts a range of functions in the gastrointestinal tract, acting as a pro-inflammatory stimulus, stimulating mucus secretion, opening chloride channels in the colon and promoting vasodilation (Brown et al. 1992; Tamai and Gaginella 1993; Fan et al. 1998). In addition, other authors have shown that citrulline alone induces endothelial relaxation as it enhances the production of NO, probably due to its recycling into arginine (Raghavan and Dikshit 2001). Particularly in the explant model described here, this mechanism would explain why *B. hyodysenteriae* exposed explants had a greater degree of necrosis than the LPS group, even though the TNF-α mRNA levels detected were lower. However, the data presented here is limited to a single sampling time point (8 h), limiting conclusions from this study. In light of the lack of evidence of toxin and tissue invasion damage by *B. hyodysenteriae*, it is hypothesized that an indirect mechanism leading to overproduction of NO may play a role in the pathogenesis of swine dysentery, especially in regards to tissue inflammation and luminal leakage of red blood cells. By focusing on the metabolic profile during infection, this study lacked further evidence of the hypothesis. The authors warrant further investigations to conclusively associate *B. hyodysenteriae* with the increased expression of iNOS and the production of NO in the swine colon.

## Conclusions

Herein, we have presented data demonstrating that *B. hyodysenteriae* induces a pro-inflammatory response in porcine colon explants, affecting different metabolic pathways that culminates with crypt epithelial necrosis. We also observed a tissular *B. hyodysenteriae*-induced accumulation of citrulline that may have resulted from the synthesis of NO, which in turn leads to vasodilation, mucus secretion, opening of chloride channels and inflammation. It is postulated that this mechanism may play a role in the pathophysiology of mucohaemorrhagic diarrhea in vivo. Further research to characterize this hypothesis is warranted, which will contribute to our ability to treat and prevent clinical disease and its consequent economic impact to producers.

## Electronic supplementary material

Below is the link to the electronic supplementary material

Supplementary Figure 1—Principal component analysis (PCA) and orthogonal partial least squares (oPLS-DA) analysis of metabolomics data (TIF 6503 KB)

Supplementary Table 1—Integrated enrichment and pathway topography analysis results (PDF 26 KB)

Supplementary Table 2—ANOVA analysis of individual metabolite concentration differences across groups (PDF 18 KB)

Supplementary Table 3—Raw LC/MS data (PDF 131 KB)
