# Supplementary figures and images for: Metabolic response of porcine colon explants to in vitro infection by Brachyspira hyodysenteriae: a leap into disease pathophysiology
Source: Metabolomics. 2017 May 30;13(7):83. doi: 10.1007/s11306-017-1219-6 (PMC5486615; doi:10.1007/s11306-017-1219-6)

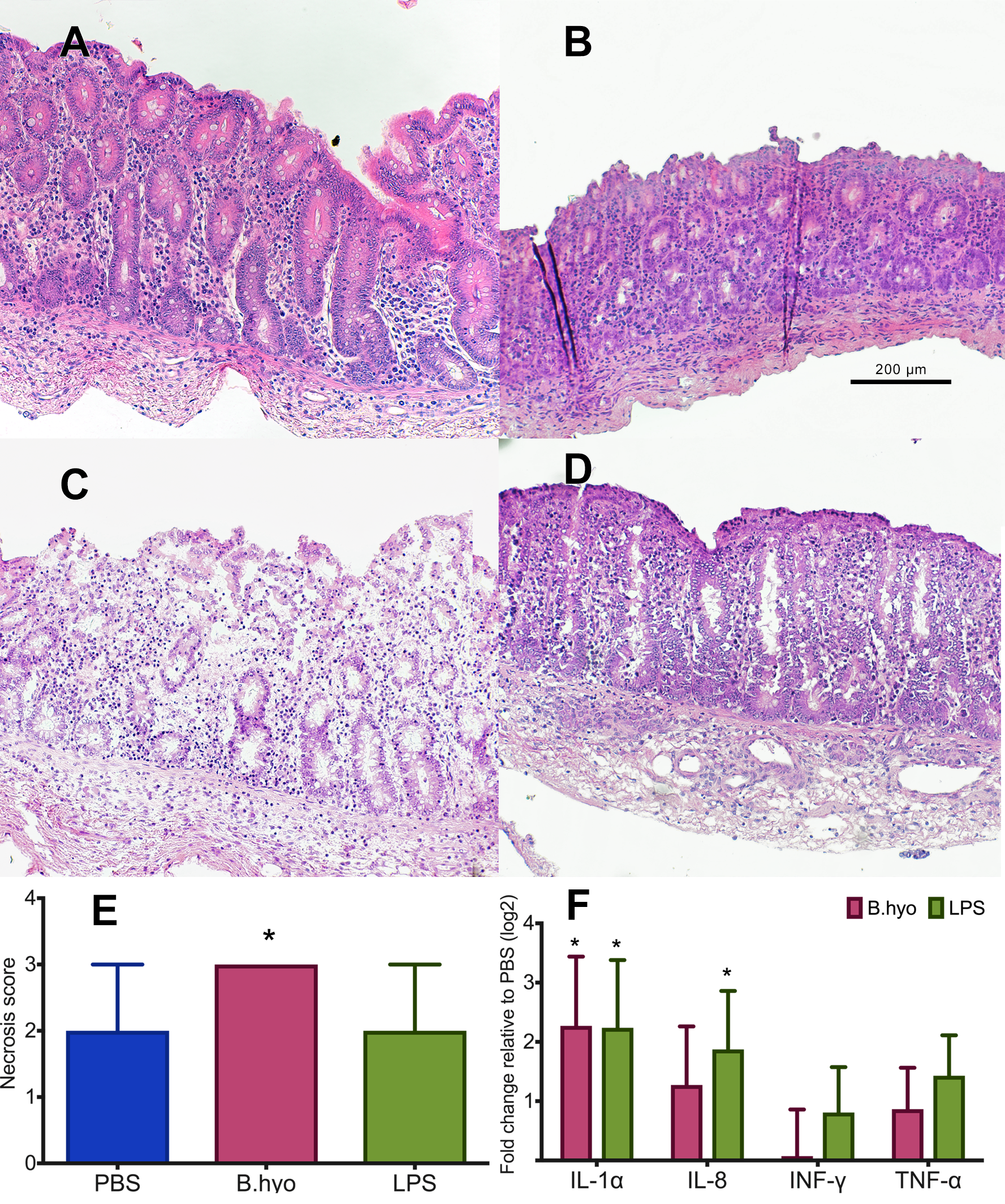

Supplement: Supplementary file 1 — Supplementary Figure 1—Principal component analysis (PCA) and orthogonal partial least squares (oPLS-DA) analysis of metabolomics data (TIF 6503 KB) [file 11306_2017_1219_MOESM1_ESM.tif]
