# Supplementary material for: Metabolic response of porcine colon explants to in vitro infection by Brachyspira hyodysenteriae: a leap into disease pathophysiology
Source: Metabolomics. 2017 May 30;13(7):83. doi: 10.1007/s11306-017-1219-6 (PMC5486615; doi:10.1007/s11306-017-1219-6)
Supplement: Supplementary file 2 — Supplementary Table 1—Integrated enrichment and pathway topography analysis results (PDF 26 KB) [file 11306_2017_1219_MOESM2_ESM.pdf]

Compounds	Total Compounds in the pathway	Hits	Raw p	LOG(p)	Holm adjust	FDR	Impact
Alanine, aspartate and glutamate metabolism	24	10	0.0019067	6.2624	0.10296	0.018749	0.75404
Synthesis and degradation of ketone bodies	6	3	0.80738	0.21396	1	0.8213	0.7
Pyruvate metabolism	32	5	0.45821	0.78043	1	0.55172	0.56886
Cysteine and methionine metabolism	56	10	0.053073	2.9361	1	0.12043	0.54736
Arginine and proline metabolism	77	13	0.0014296	6.5504	0.080058	0.018749	0.46129
Pyrimidine metabolism	60	14	0.019649	3.9297	0.91812	0.082806	0.43845
Citrate cycle (TCA cycle)	20	8	0.033995	3.3815	1	0.098086	0.33318
Glycolysis or Gluconeogenesis	31	7	0.29362	1.2255	1	0.42253	0.29508
beta-Alanine metabolism	28	6	0.34127	1.0751	1	0.44745	0.26813
Fatty acid elongation in mitochondria	27	1	0.48352	0.72665	1	0.55937	0.26765
Purine metabolism	92	12	0.26582	1.325	1	0.39658	0.26548
Butanoate metabolism	40	8	0.0010913	6.8204	0.063295	0.018749	0.26263
Pantothenate and CoA biosynthesis	27	7	0.3122	1.1641	1	0.42962	0.253
Pentose phosphate pathway	32	6	0.33617	1.0901	1	0.44745	0.2475
Galactose metabolism	41	4	0.039593	3.2291	1	0.10156	0.22669
Aminoacyl-tRNA biosynthesis	75	18	0.057098	2.863	1	0.12095	0.22536
Biotin metabolism	11	2	0.31311	1.1612	1	0.42962	0.20325
Glycine, serine and threonine metabolism	48	10	0.028604	3.5542	1	0.098086	0.19432
Glycerolipid metabolism	32	2	0.17907	1.72	1	0.30186	0.18847
Fatty acid metabolism	50	2	0.35851	1.0258	1	0.45983	0.17559
Propanoate metabolism	35	6	0.015839	4.1453	0.77609	0.078189	0.16822
Lysine degradation	47	2	0.60553	0.50165	1	0.68705	0.14675
Riboflavin metabolism	21	1	0.83265	0.18315	1	0.83265	0.14504
Histidine metabolism	44	3	0.14156	1.955	1	0.24565	0.14039
D-Glutamine and D-glutamate metabolism	11	2	0.057401	2.8577	1	0.12095	0.13904
Thiamine metabolism	24	3	0.70785	0.34553	1	0.74577	0.12481
Vitamin B6 metabolism	32	3	0.0013545	6.6043	0.077208	0.018749	0.1229
Phenylalanine metabolism	45	5	0.0052397	5.2515	0.26723	0.034349	0.11906
Tryptophan metabolism	79	2	0.027679	3.5871	1	0.098086	0.10853
Methane metabolism	34	3	0.01001	4.6042	0.5005	0.059059	0.10367
Lysine biosynthesis	32	3	0.74138	0.29925	1	0.76739	0.09993
Valine, leucine and isoleucine degradation	40	5	0.26887	1.3135	1	0.39658	0.0713
Valine, leucine and isoleucine biosynthesis	27	5	0.2159	1.5329	1	0.34428	0.06148
Amino sugar and nucleotide sugar metabolism	88	7	0.0009611	6.9474	0.056705	0.018749	0.05792
Nicotinate and nicotinamide metabolism	44	8	0.037366	3.287	1	0.10021	0.05642
Taurine and hypotaurine metabolism	20	4	0.032694	3.4206	1	0.098086	0.05395
Tyrosine metabolism	76	5	0.0034892	5.6581	0.18493	0.029409	0.04724
Fructose and mannose metabolism	48	1	0.62377	0.47197	1	0.69439	0.04115
Glyoxylate and dicarboxylate metabolism	50	6	0.050386	2.988	1	0.11891	0.03375
Sulfur metabolism	18	2	0.034912	3.3549	1	0.098086	0.03307
Inositol phosphate metabolism	39	3	0.20728	1.5737	1	0.33971	0.02406
Glutathione metabolism	38	6	0.028608	3.5541	1	0.098086	0.02214
Fatty acid biosynthesis	49	1	0.48352	0.72665	1	0.55937	0.0218
Glycerophospholipid metabolism	39	2	0.65109	0.42911	1	0.71138	0.0212
Starch and sucrose metabolism	50	1	0.13931	1.9711	1	0.24565	0.01703
Ascorbate and aldarate metabolism	45	1	0.2454	1.4049	1	0.38102	0.01617
Nitrogen metabolism	39	10	0.12011	2.1193	1	0.22815	0.0083
Phenylalanine, tyrosine and tryptophan biosynthesis	27	5	0.083361	2.4846	1	0.16394	0.008
Selenoamino acid metabolism	22	1	0.0018255	6.3059	0.1004	0.018749	0
Sphingolipid metabolism	25	1	0.0051153	5.2755	0.266	0.034349	0
Porphyrin and chlorophyll metabolism	104	1	0.015903	4.1413	0.77609	0.078189	0
D-Arginine and D-ornithine metabolism	8	2	0.019534	3.9356	0.91812	0.082806	0
One carbon pool by folate	9	2	0.03487	3.3561	1	0.098086	0
Glycosylphosphatidylinositol(GPI)-anchor biosynthesis	14	1	0.042071	3.1684	1	0.10343	0
Cyanoamino acid metabolism	16	3	0.069911	2.6605	1	0.14223	0
Folate biosynthesis	42	1	0.12374	2.0896	1	0.22815	0
Terpenoid backbone biosynthesis	33	2	0.39957	0.91738	1	0.50158	0
Pentose and glucuronate interconversions	53	2	0.45333	0.79113	1	0.55172	0
Ubiquinone and other terpenoid-quinone biosynthesis	36	1	0.68587	0.37707	1	0.73575	0
