# Supplementary material for: Metabolic response of porcine colon explants to in vitro infection by Brachyspira hyodysenteriae: a leap into disease pathophysiology
Source: Metabolomics. 2017 May 30;13(7):83. doi: 10.1007/s11306-017-1219-6 (PMC5486615; doi:10.1007/s11306-017-1219-6)
Supplement: Supplementary file 3 — Supplementary Table 2—ANOVA analysis of individual metabolite concentration differences across groups (PDF 18 KB) [file 11306_2017_1219_MOESM3_ESM.pdf]

Compound	f.value	p.value	LOG10(p)	FDR	Fisher's LSD
Pyridoxine	100.48	2.04E-18	17.691	2.42E-16	Bhyo - LPS; PBS - Bhyo; PBS - LPS
Citrulline	16.966	2.25E-06	5.6482	0.00013375	Bhyo - LPS; Bhyo - PBS
Cytosine	15.029	7.39E-06	5.1313	0.00029316	Bhyo - LPS; Bhyo - PBS
D-Glucose 6-phosphate	11.981	5.43E-05	4.2654	0.0016145	Bhyo - LPS; PBS - LPS
N-Acetyl-D-Glucosamine 6-Phosphate	10.865	0.00011728	3.9308	0.0024429	LPS - Bhyo; PBS - Bhyo
N-acetylglucosamine/N-acetylmannosamine	10.796	0.00012317	3.9095	0.0024429	LPS - Bhyo; PBS - Bhyo
Succinic acid	10.083	0.00020422	3.6899	0.0034717	PBS - Bhyo; PBS - LPS
5-Methyltetrahydrofolic acid	8.638	0.00058776	3.2308	0.008743	LPS - Bhyo; LPS - PBS
Oxidized glutathione	8.3209	0.00074566	3.1275	0.0098593	LPS - Bhyo; PBS - Bhyo
Creatine	7.8897	0.0010343	2.9854	0.012308	LPS - Bhyo; PBS - Bhyo
L-Alanine	6.8593	0.0023004	2.6382	0.024886	PBS - Bhyo; PBS - LPS
L-Arginine	5.9513	0.0047532	2.323	0.04351	LPS - Bhyo; PBS - Bhyo
Guanosine monophosphate	5.4032	0.0074422	2.1283	0.06242	PBS - Bhyo
D-Glucose 6-phosphate	4.466	0.01632	1.7873	0.12138	Bhyo - LPS; Bhyo - PBS
L-Serine	4.3258	0.018394	1.7353	0.12876	PBS - Bhyo
N-Acetylglutamic acid	4.1465	0.021451	1.6686	0.13101	Bhyo - LPS; PBS - LPS
L-Dihydroorotic acid	4.1417	0.021539	1.6668	0.13101	PBS - LPS
L-Glutamic acid	4.1162	0.022018	1.6572	0.13101	LPS - Bhyo; PBS - Bhyo
D-Malic acid	4.0136	0.024056	1.6188	0.13632	PBS - Bhyo; PBS - LPS
Myo-Inositol	3.6454	0.033132	1.4798	0.17759	LPS - Bhyo
L-Acetylcarnitine	3.605	0.034324	1.4644	0.17759	LPS - Bhyo; PBS - Bhyo
